# Outcomes in Patients With Tumor Lysis Syndrome and Comorbid Gout: A Propensity-Matched Study From a Global Federated Health Research Network

**DOI:** 10.7759/cureus.92433

**Published:** 2025-09-16

**Authors:** Sarah Eidbo, Arica Jacob, Muluken Megiso, Nanuka Tsibadze, Diego Lema Rodriguez, Justin Lam, Fabian Rodriguez Quinonez

**Affiliations:** 1 Internal Medicine, Jefferson Einstein Philadelphia Hospital, Philadelphia, USA; 2 Internal Medicine, Einstein Healthcare Network, Philadelphia, USA; 3 Rheumatology, Jefferson Einstein Philadelphia Hospital, Philadelphia, USA

**Keywords:** cohort, database, gout, tumor lysis syndrome, urate lowering therapy

## Abstract

Introduction

Tumor lysis syndrome (TLS) is an oncologic emergency that can result in cardiac arrhythmias, acute kidney injury (AKI), seizures, and death. Uric acid released in TLS can be managed with gout medications. We used a national database to study the impact of gout on patients with TLS.

Methods

The TriNetX US Collaborative Network was used for ICD-10 (International Classification of Diseases) code data. Adults with TLS were divided according to gout diagnosis. Uric acid-lowering therapies (ULTs), including allopurinol, febuxostat, and rasburicase, were studied. Cohorts were propensity-matched based on age, sex, race, and comorbidities. Rates of mortality, AKI, seizures, atrial fibrillation (AF), atrial flutter (AFL), Torsade de Pointes (TP), continuous renal replacement therapy (CRRT), septic arthritis, sepsis, intensive care unit (ICU) admission, and hospital admission were tracked for five years.

Results

TLS without gout demonstrated a higher risk of mortality (HR: 1.146; 95% CI: 1.065-1.233; p = 0.0038), AKI (HR: 1.02; 95% CI: 0.956-1.089; p = 0.0017), and sepsis (HR: 1.014; 95% CI: 0.926-1.111; p = 0.0158). TLS without allopurinol had higher risks of AKI (HR: 1.489; 95% CI: 1.337-1.658; p = 0.000), AF/AFL (HR: 1.046; 95% CI: 0.886-1.235; p = 0.002), and sepsis (HR: 1.375; 95% CI: 1.188-1.591; p = 0.027). TLS without rasburicase had a higher risk of mortality (HR: 1.165; 95% CI: 0.747-1.818; p = 0.024).

Conclusion

Patients with TLS have a higher risk of mortality, AKI, and sepsis than patients with TLS and gout. Gout and ULTs could be protective in TLS. Further research is warranted.

## Introduction

Tumor lysis syndrome (TLS)

TLS is one of a small number of oncologic emergencies. Its severity can range from mild to fatal. TLS occurs when cytotoxic chemotherapy induces massive cell lysis, leading to characteristic metabolic derangements from spilled intracellular contents [1]. These metabolic derangements can include hyperkalemia, hyperphosphatemia, hyperuricemia, hypocalcemia, and uremia [1]. Without proper management, these derangements can cause neurological, cardiac, and renal sequelae, presenting as seizures, cardiac arrhythmias, renal failure, and death [1,2].

TLS can be further subdivided into laboratory TLS and clinical TLS [1]. Laboratory TLS is defined in adults as two or more of the following laboratory values within three to seven days of initiating cytotoxic therapy: uric acid >8 mg/dL, potassium >6 mEq/L, phosphate >4.5 mg/dL, or calcium 1.5 times the upper limit of normal; cardiac arrhythmias, seizures, or death [1,2]. Clinical TLS, however, must meet the criteria for laboratory TLS and also have additional renal insufficiency, with creatinine at or above 1.5 times the upper limit of normal, seizures, cardiac arrhythmias, or sudden death [1].

The mechanism by which each electrolyte disturbance can lead to downstream sequelae is well established. When cells lyse en masse, the freed nucleic acids are catabolized to uric acid, which precipitates into crystals when overly concentrated in the kidneys, leading to renal obstruction and uropathy [3]. Similar to the sequelae following hyperuricemia, renal tubular excretion pathways for phosphate also become overwhelmed in TLS [4]. The excess phosphate binds calcium, which would normally serve to stabilize cardiac membranes [4]. This effect is compounded by hyperkalemia, which is also normally renally excreted and has excitatory effects on cardiac membranes [5]. 

Gout

Gout Pathophysiology

Gout is one of the most common forms of inflammatory arthritis in the United States, characterized by excess uric acid, leading to hyperuricemia that causes uric acid deposition in tissues and joints in the form of monosodium urate monohydrate crystals [6,7]. These crystals can trigger an acute gout flare [7]. Treatment is broken into two phases: first, reducing symptoms and inflammation of an acute flare; followed by reducing serum uric acid levels to prevent further flares and allow resolution of tophi [8-10]. Acute flares are managed with non-steroidal anti-inflammatory drugs (NSAIDs), systemic glucocorticoids, and colchicine [8-10]. The second phase of treatment, occurring after the acute flare is managed, can be achieved with uric acid-lowering therapies (ULTs): xanthine oxidase inhibitors (XOIs), uricosuric drugs, and pegloticases [8-10]. This study focused on the XOIs allopurinol and febuxostat, as well as rasburicase, a nonpegylated recombinant uricase used to prevent uric acid nephropathy in TLS.

TLS and gout

Despite many clear differences in mechanism and pathophysiology, TLS and gout share one notable common denominator: hyperuricemia. The acuity and severity of hyperuricemia may differ, but it remains a notable commonality that has yet to be explored. There is a paucity of research on patient populations diagnosed with both TLS and comorbid gout, and thus, there is little information on how these conditions may impact each other. This study aimed to determine whether patients with TLS had differences in outcomes depending on the presence of a comorbid gout diagnosis. It also aimed to elucidate any outcome differences among commonly used ULTs.

This article was previously presented as a meeting abstract in the Journal of the National Comprehensive Cancer Network on March 28, 2025, following its presentation at the 2025 NCCN National Conference. 

## Materials and methods

Two main steps were followed in this retrospective analysis: defining cohorts and analyzing cohorts. Cohort definitions were set using query criteria based on ICD-10 (International Classification of Diseases) codes. Analyses were conducted on propensity-matched cohorts to determine differences in outcomes within predefined time frames and criteria.

Defining cohorts

Eligibility Criteria

For this study, TriNetX’s US Collaborative Network - a nationwide database of deidentified health data spanning multiple large healthcare organizations (HCOs) - was used to compile patients according to specific ICD-10 codes. These codes included those related to TLS and gout, as well as codes for specific uric acid-lowering agents, including allopurinol, febuxostat, and rasburicase. The relevant codes are listed in Tables 1-2, respectively. 

**Table 1:** ICD-10 Codes for Tumor Lysis Syndrome and Gout International Classification of Diseases (ICD)-10 codes are used to identify cases of tumor lysis syndrome and gout.

Condition	ICD-10 Code
Tumor lysis syndrome	UMLS:ICD10CM:E88.3
Gout	UMLS:ICD10CM:M10
	UMLS:ICD10CM:M10.9

**Table 2:** ICD-10 Codes for Urate-Lowering Therapy Medications International Classification of Diseases (ICD)-10 codes are utilized for urate-lowering therapies (ULTs) studied.

Urate-Lowering Therapy	ICD-10 Code
Allopurinol	NLM:RXNORM:519
Febuxostat	NLM:RXNORM:73689
Rasburicase	NLM:RXNORM:283821

Cohort Definitions

For each uric acid-lowering medication studied, two cohorts were compared: a cohort of patients diagnosed with TLS and gout who were prescribed the medication in question, and a cohort of patients with TLS and no gout. One cohort, consisting of patients diagnosed with TLS and comorbid gout and on any of the three medications listed above, was also compared to a cohort of patients with TLS and without gout.

For the TLS without gout cohort, a query was run on 142 HCOs, with 22,357 patients matching the query criteria. For the TLS with gout on allopurinol cohort, a query was run on 142 HCOs, with 981 patients matching the criteria. For the TLS with gout on febuxostat cohort, a query was run on 142 HCOs, with 31 patients matching the query criteria. For the TLS with gout on rasburicase cohort, a query was run on 142 HCOs, with 68 patients matching the query criteria. For the TLS with gout on any medication cohort, a query was run on 142 HCOs, with 2,462 patients matching the query criteria.

Statistical analysis

Index events and time windows were defined in order to analyze cohorts. The day on which a patient first met eligibility criteria for a cohort was defined as the index event. Outcomes were followed for five years after the index event. These outcomes included rates of all-cause mortality; acute kidney injury (AKI); atrial fibrillation (AF) or atrial flutter (AFL); ventricular fibrillation (VF), ventricular tachycardia (VT), or Torsade de Pointes (TP); seizures; continuous renal replacement therapy (CRRT); sepsis; septic arthritis; and admission to intensive care units (ICUs). Outcome ICD-10 codes are listed in Table 3. 

**Table 3:** ICD-10 Codes for Outcomes International Classification of Diseases (ICD-10) codes used to identify outcomes are listed here. Outcomes included rates of all-cause mortality; acute kidney injury (AKI); atrial fibrillation (AF) or atrial flutter (AFL); ventricular fibrillation (VF), ventricular tachycardia (VT), or Torsade de Pointes (TP); seizures; continuous renal replacement therapy (CRRT); sepsis; septic arthritis; and admission to intensive care units (ICUs).

Outcome	Name	ICD-10 Code
All-Cause Mortality	Deceased	Deceased
Acute Kidney Injury (AKI)	Acute kidney failure	UMLS:ICD10CM:N17
	Acute kidney failure, unspecified	UMLS:ICD10CM:N17.9
Atrial Fibrillation (AF) or Atrial Flutter (AFL)	Unspecified atrial fibrillation	UMLS:ICD10CM:I48.91
	Paroxysmal atrial fibrillation	UMLS:ICD10CM:I48.0
	Persistent atrial fibrillation	UMLS:ICD10CM:I48.1
	Chronic atrial fibrillation	UMLS:ICD10CM:I48.2
	Other persistent atrial fibrillation	UMLS:ICD10CM:I48.19
	Permanent atrial fibrillation	UMLS:ICD10CM:I48.21
	Atrial fibrillation and flutter	UMLS:ICD10CM:I48
	Chronic atrial fibrillation, unspecified	UMLS:ICD10CM:I48.20
	Longstanding persistent atrial fibrillation	UMLS:ICD10CM:I48.11
	Unspecified atrial fibrillation and atrial flutter	UMLS:ICD10CM:I48.9
Ventricular Fibrillation (VF) or Ventricular Tachycardia (VT) or Torsade de Pointes (TP)	Ventricular fibrillation	UMLS:ICD10CM:I49.01
	Ventricular fibrillation and flutter	UMLS:ICD10CM:I49.0
	Ventricular tachycardia	UMLS:ICD10CM:I47.2
	Ventricular tachycardia, unspecified	UMLS:ICD10CM:I47.20
	Other ventricular tachycardia	UMLS:ICD10CM:I47.29
	Long QT syndrome	UMLS:ICD10CM:I45.81
	Ventricular fibrillation	UMLS:ICD10CM:I49.01
	Torsades de Pointes	UMLS:ICD10CM:I47.21
Seizures	Convulsions, not elsewhere classified	UMLS:ICD10CM:R56
	Other seizures	UMLS:ICD10CM:G40.89
	Epilepsy, unspecified, intractable, without status epilepticus	UMLS:ICD10CM:G40.919
Continuous Renal Replacement Therapy (CRRT)	Continuous renal replacement therapy	UMLS:SNOMED:714749008
	Prolonged intermittent renal replacement therapy	UMLS:SNOMED:895382009
	Dialysis procedure other than hemodialysis (eg, peritoneal dialysis, hemofiltration, or other continuous renal replacement therapies), with single evaluation by a physician or other qualified health care professional	UMLS:CPT:90945
	Dialysis procedure other than hemodialysis (eg, peritoneal dialysis, hemofiltration, or other continuous renal replacement therapies) requiring repeated evaluations by a physician or other qualified health care professional, with or without substantial revision of dialysis prescription	UMLS:CPT:90947
	Hemodialysis procedures	UMLS:CPT:1012752
	Hemodialysis	UMLS:SNOMED:302497006
	Hemodialysis procedure with a single evaluation by a physician or other qualified health care professional	UMLS:CPT:90935
	Hemodialysis	UMLS:ICD9CM:39.95
	Dependence on renal dialysis	UMLS:ICD10CM:Z99.2
Sepsis	Other specified sepsis	UMLS:ICD10CM:A41.89
	Sepsis, unspecified organism	UMLS:ICD10CM:A41.9
	Sepsis due to *Escherichia coli* (*E. coli*)	UMLS:ICD10CM:A41.51
	Sepsis due to other Gram-negative organisms	UMLS:ICD10CM:A41.5
	Sepsis due to *Staphylococcus aureus*	UMLS:ICD10CM:A41.0
	Sepsis due to Methicillin-susceptible *Staphylococcus aureus*	UMLS:ICD10CM:A41.01
	Sepsis due to Methicillin-resistant *Staphylococcus aureus*	UMLS:ICD10CM:A41.02
	Sepsis due to other specified *Staphylococcus*	UMLS:ICD10CM:A41.1
	Sepsis due to *Pseudomonas*	UMLS:ICD10CM:A41.52
	Sepsis due to *Enterococcus*	UMLS:ICD10CM:A41.81
	Sepsis due to anaerobes	UMLS:ICD10CM:A41.4
	Sepsis due to *Streptococcus pneumoniae*	UMLS:ICD10CM:A40.3
	Sepsis due to unspecified *Staphylococcus*	UMLS:ICD10CM:A41.2
	Sepsis due to *Streptococcus*, group A	UMLS:ICD10CM:A40.0
	Sepsis due to *Streptococcus*, group B	UMLS:ICD10CM:A40.1
Septic Arthritis	Arthritis due to other bacteria, unspecified joint	UMLS:ICD10CM:M00.80
	Staphylococcal arthritis, unspecified joint	UMLS:ICD10CM:M00.00
	Arthritis due to other bacteria, unspecified knee	UMLS:ICD10CM:M00.869
	Staphylococcal arthritis, unspecified knee	UMLS:ICD10CM:M00.069
	Arthritis due to other bacteria, unspecified hip	UMLS:ICD10CM:M00.859
	Staphylococcal arthritis, unspecified hip	UMLS:ICD10CM:M00.059
	Arthritis due to other bacteria, unspecified ankle and foot	UMLS:ICD10CM:M00.879
	Staphylococcal arthritis, unspecified ankle and foot	UMLS:ICD10CM:M00.079
	Arthritis due to other bacteria, unspecified elbow	UMLS:ICD10CM:M00.829
	Staphylococcal arthritis, unspecified elbow	UMLS:ICD10CM:M00.029
	Arthritis due to other bacteria, unspecified wrist	UMLS:ICD10CM:M00.839
	Staphylococcal arthritis, unspecified wrist	UMLS:ICD10CM:M00.039
	Arthritis due to other bacteria, unspecified shoulder	UMLS:ICD10CM:M00.819
	Staphylococcal arthritis, unspecified shoulder	UMLS:ICD10CM:M00.019
	Arthritis due to other bacteria, unspecified hand	UMLS:ICD10CM:M00.849
	Staphylococcal arthritis, unspecified hand	UMLS:ICD10CM:M00.049
	Arthritis due to other bacteria, vertebrae	UMLS:ICD10CM:M00.88
	Staphylococcal arthritis, vertebrae	UMLS:ICD10CM:M00.08
Intensive Care Unit Admission (ICU)	Admission to intensive care unit	UMLS:SNOMED:305351004
	Intensive care monitoring	UMLS:SNOMED:182810003

Kaplan-Meier analyses were used to calculate hazard ratios, log-rank tests, and proportionality tests, alongside median survival (defined as the number of days before cohort survival drops below 50%) and survival probability (the percent of the cohort that survives until the time window ends). Patients who exited a cohort during the analysis time window were removed from the analysis.

Propensity matching for cohorts was performed according to various demographics and diagnostic codes. ICD-10 codes used for propensity matching according to demographics and diagnoses are listed in Tables 4-5, respectively. 

**Table 4:** ICD-10 Codes for Propensity-Score Matching Demographics International Classification of Diseases (ICD-10) codes are utilized to propensity-score match cohorts according to demographics.

Demographic	ICD-10 Code
Current Age	Age
White Patients	2106-3
Unknown Race	UNK
Female	F
Native Hawaiian or Other Pacific Islander Patients	2076-8
Unknown Ethnicity	UNK
Not Hispanic or Latino Patients	2186-5
Hispanic or Latino Patients	2135-2
Black or African American Patients	2054-5
Male	M
Other Race	2131-1
Asian Patients	2028-9

**Table 5:** ICD-10 Codes for Propensity-Score Matching Diagnoses International Classification of Diseases (ICD-10) codes are utilized for propensity-score matching cohorts according to preexisting diagnoses.

Diagnosis	ICD-10 Code
Neoplasms	C00-D49
Symptoms, signs and abnormal clinical and laboratory findings, not elsewhere classified	R00-R99
Endocrine, nutritional and metabolic diseases	E00-E89
Factors influencing health status and contact with health services	Z00-Z99
Diseases of the circulatory system	I00-I99
Diseases of the blood and blood-forming organs and certain disorders involving the immune mechanism	D50-D89
Diseases of the digestive system	K00-K95
Diseases of the musculoskeletal system and connective tissue	M00-M99
Diseases of the genitourinary system	N00-N99
Diseases of the respiratory system	J00-J99
Diseases of the nervous system	G00-G99

## Results

Allopurinol

After propensity-score matching, 981 patients were identified in each cohort diagnosed with TLS - with and without gout - and prescribed allopurinol. The average age at index for patients with gout was 72.9 ± 12.2 years, compared to 64.9 ± 18.3 years for patients without gout. In the gout cohort, 22.3% (n = 219) were female, compared to 37.4% (n = 367) in the cohort without gout. The ethnic distribution in the gout cohort was predominantly Caucasian patients (70.7%, n = 694), followed by Black/African American patients (16.2%, n = 159), Asian patients (3.6%, n = 35), Hispanic/Latino patients (3.0%, n = 29), and Native Hawaiian/Pacific Islander patients (1.0%, n = 10). This is compared to the ethnic distribution in the non-gout cohort, which remained predominantly Caucasian patients (64.3%, n = 631), followed by Black/African American patients (12.9%, n = 127), Hispanic/Latino patients (7.8%, n = 77), Asian patients (4.3%, n = 42), and Native Hawaiian/Pacific Islander patients (0.7%, n = 7). The TLS without gout cohort demonstrated a significantly higher risk of AKI (HR: 1.489, 95% CI: 1.337-1.658, p = 0.000), AF/AFL (HR: 1.046, 95% CI: 0.886-1.235, p = 0.002), and sepsis (HR: 1.375, 95% CI: 1.188-1.591, p = 0.027). The cohorts demonstrated no significant differences in rates of mortality (HR: 1.541, 95% CI: 1.368-1.735, p = 0.109), VT/VF/TP (HR: 1.195, 95% CI: 0.901-1.585, p = 0.147), seizures (HR: 1.376, 95% CI: 0.938-2.018, p = 0.419), CRRT (HR: 1.659, 95% CI: 1.277-2.154, p = 0.834), septic arthritis (HR: 0.527, 95% CI: 0.102-2.723, p = 0.640), or ICU admission (no patients in either cohort met criteria for this outcome).

Febuxostat

After propensity-score matching, 31 patients were identified in each cohort who were diagnosed with TLS - with and without gout - on febuxostat. The average age at index for the TLS and gout cohort was 70 ± 12.2 years, compared to 64.9 ± 18.3 years for patients with TLS without gout. In the TLS and gout cohort, 31.3% (n = 10) were female, compared to 37.4% (n = 12) in the cohort without gout. Within the TLS and gout cohort, the ethnic distribution was predominantly Asian patients (75.0%, n = 21), followed by Caucasian patients (31.3%, n = 10) and Native Hawaiian/Pacific Islander patients (31.3%, n = 10), with no patients in the African American or Hispanic/Latino categories (0%). In the TLS cohort without gout, the cohort was predominantly Caucasian patients (64.3%, n = 20), followed by African American patients (12.9%, n = 4), Hispanic/Latino patients (7.8%, n = 2), Asian patients (4.3%, n = 1), and Native Hawaiian/Pacific Islander patients (0.7%, n = 1). The two cohorts demonstrated no significant differences between rates of mortality (HR: 1.657, 95% CI: 0.762-3.599, p = 0.909), AKI (HR: 2.169, 95% CI: 1.170-4.022, p = 0.917), AF/AFL (HR: 2.107, 95% CI: 0.720-6.166, p = 0.496), VT/VF/TP (HR: 2.972, 95% CI: 0.309-28.573, p = 0.273), seizures (no patients in one cohort had this outcome; HR, 95% CI, and p-value unable to be calculated), CRRT (HR: 1.257, 95% CI: 0.519-3.044, p = 0.129), septic arthritis (no patients in either cohort met criteria for this outcome), sepsis (HR: 1.192, 95% CI: 0.526-2.704, p = 0.371), or ICU admission (no patients in either cohort met criteria for this outcome).

Rasburicase

After propensity-score matching, 68 patients were identified in each cohort with TLS - with and without gout - on rasburicase. The average age at index for patients with TLS and gout was 72.1 ± 11.7 years, compared to the TLS without gout cohort, 64.9 ± 18.3 years. In the TLS with gout and rasburicase cohort, 20.6% (n = 14) were female, compared to 37.5% (n = 26) female within the cohort without gout. The ethnic distribution within the cohort with TLS and gout on rasburicase was predominantly Caucasian patients (48.5%, n = 32), followed by African American patients, Asian patients, Hispanic/Latino patients, and Native Hawaiian/Pacific Islander patients, all equally distributed (14.7%, n = 9). Within the TLS without comorbid gout cohort, the patients were predominantly Caucasian patients (64.3%, n = 44), followed by African American patients (12.9%, n = 8), Hispanic/Latino patients (7.8%, n = 5), Asian patients (4.3%, n = 3), and Native Hawaiian/Pacific Islander patients (0.7%, n = 1). The TLS without gout cohort demonstrated a significantly higher risk of mortality (HR: 1.165, 95% CI: 0.747-1.818, p = 0.024). There was no significant difference between rates of AKI (HR: 1.011, 95% CI: 0.678-1.509, p = 0.577), AF/AFL (HR: 1.463, 95% CI: 0.698-3.064, p = 0.139), VT/VF/TP (HR: 2.163, 95% CI: 0.666-7.027, p = 0.312), seizures (HR: 1.650, 95% CI: 0.394-6.913, p = 0.345), CRRT (HR: 0.298, 95% CI: 0.107-0.828, p = 0.648), septic arthritis (no patients in one cohort had this outcome; HR, 95% CI, and p-value unable to be calculated), sepsis (HR: 1.090, 95% CI: 0.614-1.936, p = 0.060), or ICU admission (no patients in one cohort had this outcome; HR, 95% CI, and p-value unable to be calculated).

Any ULT

After propensity-score matching, 2,462 patients were identified in each cohort with TLS - with and without gout - on any ULT. The average age at index for patients with TLS and gout was 73.0 ± 12.1 years, compared to 64.9 ± 18.3 years for the cohort without gout. Approximately 22.6% (n = 556) of the TLS with gout cohort patients were female, compared to 37.5% (n = 923) female in the cohort without gout. Within the TLS and gout cohort, ethnic distribution was predominantly Caucasian patients (64.1%, n = 1,578), followed by African American patients (15.2%, n = 374), Asian patients (6.9%, n = 170), Hispanic/Latino patients (3.6%, n = 89), and Native Hawaiian/Pacific Islander patients (2.0%, n = 49). The ethnic distribution among the cohort with TLS and without gout was Caucasian patients (64.3%, n = 1,583), followed by African American patients (12.9%, n = 318), Hispanic/Latino patients (7.8%, n = 192), Asian patients (4.3%, n = 106), and Native Hawaiian/Pacific Islander patients (0.7%, n = 172). The TLS without gout cohort demonstrated a significantly higher risk of mortality (HR: 1.329, 95% CI: 1.237-1.429, p = 0.000), AKI (HR: 1.072, 95% CI: 1.005-1.143, p = 0.001), CRRT (HR: 1.013, 95% CI: 0.877-1.171, p = 0.003), and sepsis (HR: 1.097, 95% CI: 1.005-1.198, p = 0.018). There were no significant differences between AF/AFL (HR: 0.990, 95% CI: 0.896-1.094, p = 0.087), VT/VF/TP (HR: 0.896, 95% CI: 0.750-1.070, p = 0.214), seizures (HR: 1.415, 95% CI: 1.135-1.765, p = 0.324), septic arthritis (HR: 0.373, 95% CI: 0.136-1.028, p = 0.103), or ICU admission (HR: 2.175, 95% CI: 0.196-24.070, p = 0.342).

Outcomes of patients with TLS and gout, both with and without ULTs, are summarized in Table 6.

**Table 6:** Outcomes of Patients With TLS and Gout With and Without ULTs Outcomes after propensity-score matching for each comparison - tumor lysis syndrome (TLS) without any urate-lowering therapies (ULTs) or gout compared to TLS and gout with each ULT, as listed: allopurinol, febuxostat, and rasburicase. Statistical significance is defined as a p-value at or less than p = 0.05. Cells with asterisks (*) indicate a significantly different risk of this outcome. Cells with a hyphen (-) indicate that no patients met the criteria for either cohort; thus, this value was unable to be calculated. Outcomes included rates of all-cause mortality, acute kidney injury (AKI), atrial fibrillation (AF) or atrial flutter (AFL), ventricular fibrillation (VF) or ventricular tachycardia (VT) or Torsade de pointes (TP), seizures, continuous renal replacement therapy (CRRT), sepsis, septic arthritis, and admission to intensive care units (ICUs).

p-value	Group 1	Group 2	AKI	AF/AFL	VF/VT/VP	Seizures	CRRT	Sepsis	Septic Arthritis	ICU	Mortality
	TLS without ULT	TLS with any ULT	0.001*	0.087	0.214	0.324	0.003*	0.018*	0.103	0.342	0.000*
	TLS without ULT	TLS with allopurinol	0.000*	0.002*	0.147	0.419	0.834	0.027*	0.64	-	0.109
	TLS without ULT	TLS with febuxostat	0.917	0.496	0.273	-	0.129	0.371	-	-	0.909
	TLS without ULT	TLS with rasburicase	0.577	0.139	0.312	0.345	0.648	-	0.06	-	0.024*
HR	Group 1	Group 2	AKI	AF/AFL	VF/VT/VP	Seizures	CRRT	Sepsis	Septic Arthritis	ICU	Mortality
	TLS without ULT	TLS with any ULT	1.072*	0.99	0.896	1.415	1.013*	1.097*	0.373	2.175	1.329*
	TLS without ULT	TLS with allopurinol	1.489*	1.046*	1.195	1.376	1.659	1.375*	0.527	-	1.541
	TLS without ULT	TLS with febuxostat	2.169	2.107	2.972	-	1.257	1.192	-	-	1.657
	TLS without ULT	TLS with rasburicase	1.011	1.463	2.163	1.65	0.298	-	1.09	-	1.165*
95% CIs	Group 1	Group 2	AKI	AF/AFL	VF/VT/VP	Seizures	CRRT	Sepsis	Septic Arthritis	ICU	Mortality
	TLS without ULT	TLS with any ULT	1.005 - 1.143*	0.896 - 1.094	0.750 - 1.070	1.135 - 1.765	0.877 - 1.171*	1.005 - 1.198*	0.136 - 1.028	0.196 - 24.070	1.237 - 1.429*
	TLS without ULT	TLS with allopurinol	1.337 - 1.658*	0.886 - 1.235*	0.901 - 1.585	0.938 - 2.018	1.277 - 2.154	1.188 - 1.591*	0.102 - 2.723	-	1.368 - 1.735
	TLS without ULT	TLS with febuxostat	1.170 - 4.022	0.720 - 6.166	0.309 - 28.573	-	0.519 - 3.044	0.526 - 2.704	-	-	0.762 - 3.599
	TLS without ULT	TLS with rasburicase	0.678 - 1.509	0.698 - 3.064	0.666 - 7.027	0.394 - 6.913	0.107 - 0.828	-	0.614 - 1.936	-	0.747 - 1.818*

## Discussion

Findings

In this report, we described a large, nationwide retrospective analysis of patients with TLS and gout that demonstrated multiple differences compared to their counterparts without gout. To study the effects of gout on outcomes in TLS, groups were further subdivided by ULT regimens. This allowed for assessment of any drug-specific benefit or difference. Of the medications studied, the febuxostat gout cohort was noted to have no differences in outcomes. It is possible that this finding is partially due to the limited power of this cohort, as only 31 patients were studied. The febuxostat cohort was by far the smallest in this study. As other forms of ULT were associated with benefits, it is likely that similar benefits would be seen in this cohort with a larger sample size.

The allopurinol and rasburicase cohorts were both found to have significant differences in outcomes, however. Patients with TLS and no gout had a significantly higher risk of developing AKI, AF, AFL, and sepsis compared to patients with TLS and gout who were prescribed allopurinol. These results imply that gout, and specifically allopurinol use, could be protective for patients with TLS. This unexpected finding could relate to the mechanism of allopurinol. As an XOI, allopurinol serves as a ULT - lowering levels of one metabolite that can become severely elevated in cases of TLS. It is possible that patients already on allopurinol for gout who develop TLS experience a protective buffer against higher serum uric acid levels that could correspond with poor outcomes. ULTs could be expected to protect against AKI, as hyperuricemia can lead to nephrolithiasis and other renal issues. The demonstrated protective benefit against AF/AFL and sepsis is harder to explain. Previous studies have demonstrated a possible reduction in rates of AF and AFL in elderly patients taking allopurinol, but the mechanism remains unclear [11-13]. Interestingly, XOIs may also be protective in cases of sepsis, as the condition has been demonstrated to activate xanthine oxidase [14]. In patients hospitalized with TLS, who are likely immunocompromised from chemotherapy and thus at higher risk of infection, XOI may provide additional protection. However, mechanisms are still unclear, and further research is warranted [14].

There were also significantly higher rates of mortality in the TLS cohort compared to the TLS with gout cohort that was prescribed rasburicase. Of note, rasburicase is a form of ULT that is not approved for gout management alone but rather is used for its rapid effects to protect against hyperuricemia in TLS [11]. This finding demonstrates that hyperuricemia can be associated with worse outcomes in patients with TLS, further providing evidence for the benefit of ULTs in TLS management. Though rasburicase has been established as ULT for patients at risk of developing TLS, it is mostly used in children with leukemia or lymphoma [11]. Rasburicase has not been studied extensively in adults. Previous studies have demonstrated that rasburicase may have some benefit in adults with TLS, though the benefit is unclear and may not be cost-effective [15]. Our data demonstrate a significant mortality benefit, in line with prior studies [15,16]. Further research is warranted to better define this benefit and to more thoroughly evaluate the cost. In addition to exploring specific ULTs, the impact of gout with any therapy on TLS was studied. Patients with TLS were at a significantly higher risk of mortality, AKI, CRRT, and sepsis than patients with TLS and gout. As discussed above, this implies a protective benefit of gout for patients with TLS. This finding is likely due to patients with gout already having ULT regimens. Some specific benefits of XOI are discussed above. Aside from XOI, reducing levels of hyperuricemia is protective against the development of nephrolithiasis and renal damage, and thus can potentially explain the reduction in AKI and CRRT seen in patients with TLS and gout [6,7]. A gout diagnosis and some form of ULT could also protect patients with TLS against sepsis, a state known to increase uric acid levels and likely perpetuate the negative renal effects already experienced [14]. Further research to explore mechanisms and establish other possible benefits should be conducted.

This study has several limitations that are worth acknowledging. The TriNetX nationwide database was used to collect deidentified patient data across multiple large HCOs, and it is possible that some of these HCOs did not allow access to data. In addition to possible denied access, coding errors are impossible to exclude. If patients did not receive proper diagnosis coding in their electronic records, they could have been inappropriately included or excluded from our cohorts. Results of this study should be interpreted within an appropriate context and with caution, to prevent misinterpretation or misuse.

## Conclusions

In conclusion, patients with TLS are at a significantly higher risk of mortality, AKI, CRRT, and sepsis than patients with TLS and gout on any therapy. There is a possible protective benefit of ULT, utilized in gout management, for patients with TLS that should be further explored. These outcome differences are seen among different ULT modalities, though notably not in the febuxostat cohort, which was the smallest cohort studied. Research should focus on further characterizing the benefits noted in this study, as well as determining if additional benefits or protections could exist for patients with TLS and gout.
